# Suspected Seronegative Spondyloarthropathy in a Young Woman With Ulcerative Colitis and Concurrent Bleeding Symptoms

**DOI:** 10.7759/cureus.116192

**Published:** 2026-09-13

**Authors:** Eric Walker, Shreebridhi Pande

**Affiliations:** 1 Internal Medicine, Cape Fear Valley Medical Center, Fayetteville, USA

**Keywords:** enthesitis, extraintestinal manifestations, leukopenia, seronegative spondyloarthropathy, ulcerative colitis

## Abstract

Extraintestinal musculoskeletal manifestations, including peripheral and axial spondyloarthritis, are well recognized in ulcerative colitis (UC) and are frequently associated with human leukocyte antigen B27 (HLA-B27) positivity. We present a 20-year-old woman with UC who developed inflammatory-pattern polyarthralgia involving the knees, ankles, wrists, shoulders, and lower back, with morning stiffness improving with movement, alongside bruising, menorrhagia, and mild leukopenia. Examination showed bilateral lower extremity ecchymoses and mild joint tenderness without swelling or synovitis. Laboratory evaluation showed positive HLA-B27, mildly elevated CRP, normal coagulation studies, and iron deficiency without anemia. Sacroiliac and knee radiographs were unremarkable; MRI was not obtained. von Willebrand disease, immune thrombocytopenia, and anti-neutrophil cytoplasmic antibody (ANCA)-associated vasculitis were considered less likely but not definitively excluded. Rheumatology evaluation supported a working diagnosis of inflammatory bowel disease (IBD)-associated seronegative spondyloarthropathy, with biologic therapy under consideration. This case illustrates the diagnostic complexity that arises when inflammatory musculoskeletal symptoms of IBD coexist with unexplained hematologic findings, and underscores the need for broad differential diagnosis and complete multidisciplinary evaluation before diagnostic closure.

## Introduction

Ulcerative colitis (UC) is a chronic inflammatory bowel disease (IBD) associated with multiple extraintestinal manifestations (EIMs), most commonly involving the musculoskeletal and dermatologic systems [1-3]. Peripheral arthritis and axial spondyloarthropathy may occur independently of bowel disease activity and are frequently associated with human leukocyte antigen B27 (HLA-B27) positivity [4-6]. These two musculoskeletal phenotypes, though both classified within the spondyloarthritis (SpA) family, are clinically distinct. Peripheral arthritis is typically oligoarticular and asymmetric, involving large joints in an acute, self-limited course that parallels intestinal disease activity, or less commonly polyarticular and symmetric with a more persistent course independent of bowel activity; axial spondyloarthropathy, in contrast, involves the sacroiliac joints and spine, presenting as inflammatory back or buttock pain with stiffness that improves with activity, and characteristically runs independently of intestinal inflammation [7]. The HLA-B27 association also differs by phenotype. Idiopathic ankylosing spondylitis is HLA-B27-positive in over 90% of cases; this association is weaker in IBD-associated axial disease (approximately 50-70%), while peripheral arthritis associates instead with a distinct set of HLA alleles (DRB1*0103, B27, and B58 in UC) [7]. Early recognition of these patterns is important, as musculoskeletal symptoms may precede or overshadow gastrointestinal complaints and significantly affect quality of life [1,3].

The evaluation of inflammatory symptoms in UC becomes more challenging when accompanied by unexplained bruising, menorrhagia, or hematologic abnormalities, which broaden the differential to include coagulopathies, platelet dysfunction, vasculitis, and hematologic disease [8,9]. This is compounded by IBD's own recognized vascular effects. Patients carry an approximately three-fold increased risk of venous thromboembolism, driven by endothelial dysfunction, platelet activation, and impaired fibrinolysis, with risk rising alongside inflammatory severity, as well as increased arterial risk including aortic stiffness, myocardial infarction, and cerebrovascular events [7]. Distinguishing these IBD-associated vascular and inflammatory manifestations from an unrelated coagulopathy or systemic process therefore requires careful clinical correlation and targeted evaluation.

This case is documented because it illustrates a diagnostically challenging overlap not well represented in the literature. Unlike classic IBD-associated peripheral arthritis, which typically parallels bowel activity with overt joint findings, this patient presented with an axial-predominant, entheseal pattern and only mild examination findings. The patient's pattern of bruising, menorrhagia, and leukopenia is also atypical for IBD EIMs. This case underscores the importance of maintaining a broad differential and pursuing systematic, multidisciplinary workup, rather than premature diagnostic closure, when musculoskeletal EIMs of IBD coexist with atypical systemic findings.

## Case presentation

Initial presentation

A 20-year-old woman with a history of UC, complex/vestibular migraine, and gastritis presented in December 2025 for evaluation of progressive fatigue, diffuse joint pain, bruising, and heavy menstrual bleeding. She reported approximately one month of worsening fatigue, requiring frequent daytime naps between work shifts and intermittent rest during routine activities such as showering.

She described a seven-month history of progressive polyarticular joint pain involving the bilateral knees, ankles, wrists, shoulders, and lower back, associated with morning stiffness lasting approximately 30 minutes daily that improved with movement and worsened with rest - a pattern consistent with an inflammatory arthropathy. She had obtained relief with heating pads and had not been taking scheduled medication for her joint symptoms. She was, however, already taking naproxen 500 mg PO as needed, Motrin 200 mg as needed, and acetaminophen 325 mg as needed for migraine relief prior to the onset of her joint complaints; given this existing as-needed nonsteroidal anti-inflammatory drug (NSAID) use and her history of gastritis, there was no perceived need to additionally initiate a scheduled NSAID for her musculoskeletal symptoms. Prior outpatient evaluation had demonstrated HLA-B27 positivity, and she had previously been referred to rheumatology but was unable to complete follow-up.

Her UC was diagnosed in 2021 and confirmed by colonoscopy demonstrating colitis from the rectum through the descending colon with biopsy-proven chronic inflammation. She was maintained on mesalamine 375 mg (one to two capsules daily, later increased to four capsules daily by pediatric gastroenterology) for approximately one year, then remained off IBD-directed therapy for approximately four years before a repeat colonoscopy in 2026 confirmed mild left-sided chronic colitis, at which point gastroenterology restarted mesalamine 1.2 g, two tablets daily.

She additionally reported a several-month history of progressive bruising and mildly painful ecchymotic/rash-like lesions involving the bilateral lower extremities and thighs, without associated pruritus or preceding trauma; a prior rash involving the upper extremities had resolved spontaneously. Laboratory workup obtained to evaluate for von Willebrand disease (VWD) and immune thrombocytopenic purpura, including prothrombin time (PT), international normalized ratio (INR), activated partial thromboplastin time (aPTT), complete blood count (CBC), anti-neutrophil cytoplasmic antibody (ANCA) panel, and iron panel, was unremarkable. She endorsed menorrhagia despite regular 28- to 30-day menstrual cycles, with bleeding lasting approximately five days and requiring pad changes every one to two hours during menses.

Additional history was notable for a mildly tender, non-palpable right axillary nodule present for approximately four months that had gradually decreased in size and was mildly painful with deodorant use or friction; she denied fever, unintentional weight loss, night sweats, or additional lymphadenopathy. A gynecologic evaluation approximately three months prior, including breast examination, had been unremarkable, and she had been referred to obstetrics/gynecology for further evaluation of the nodule but was unable to complete follow-up. Her migraines had been improving on amitriptyline, with a reduction from two to three episodes per week to approximately two episodes over the preceding two weeks. She was a nonsmoker and denied alcohol use. Current medications included amitriptyline 25 mg nightly, meclizine 25 mg as needed for dizziness, and sumatriptan 100 mg as needed for migraines.

Physical examination (initial presentation, December 2025)

Vital Signs

The patient’s vital signs were as follows: blood pressure, 135/92 mmHg; heart rate, 69 bpm; temperature, 37.2°C (98.9°F); respiratory rate, 18 breaths/minute; height, 65 inches (5′5″); weight, 156.6 lb; BMI, 26.06 kg/m²; and body surface area (BSA), 1.78 m²).

Physical Exam Findings

Head, ears, nose, and throat (HENT) exam showed a normocephalic head, normal nose, and moist oral mucous membranes. Eye exam showed normal conjunctivae/sclerae. Cardiovascular exam showed normal rate and regular rhythm with normal pulses. Pulmonary exam showed normal effort and breath sounds without wheezing or rhonchi. Abdominal exam showed normal bowel sounds without distension, no palpable mass, and no right or left costovertebral angle (CVA) tenderness. Musculoskeletal exam showed mild tenderness on deep palpation of the knee joints without gross joint swelling, and normal range of motion. Skin exam showed multiple bluish ecchymotic patches on the bilateral lower legs, predominantly on the extensor surfaces. Neurologic exam showed the patient alert and oriented to person, place, and time, with mental status at baseline. Psychiatric exam showed normal mood and affect.

Diagnostic imaging

Dedicated anteroposterior radiography of the bilateral sacroiliac joints (May 2026) and bilateral knees (December 2025) was performed and interpreted as unremarkable, without radiographic evidence of sacroiliitis, sclerosis, erosions, or joint space narrowing (Figure 1). Plain radiography has limited sensitivity for detecting early inflammatory changes of axial SpA, and a normal radiograph demonstrates only the absence of radiographic sacroiliitis rather than excluding active axial inflammatory disease. MRI of the sacroiliac joints, which would be needed to assess for bone marrow edema and active inflammation in suspected non-radiographic axial SpA, was not obtained and is not documented in the available records; this represents a limitation of the current diagnostic workup. Bilateral ankle radiographs were ordered at the most recent rheumatology visit, with results not available in the documentation reviewed.

**Figure 1:**
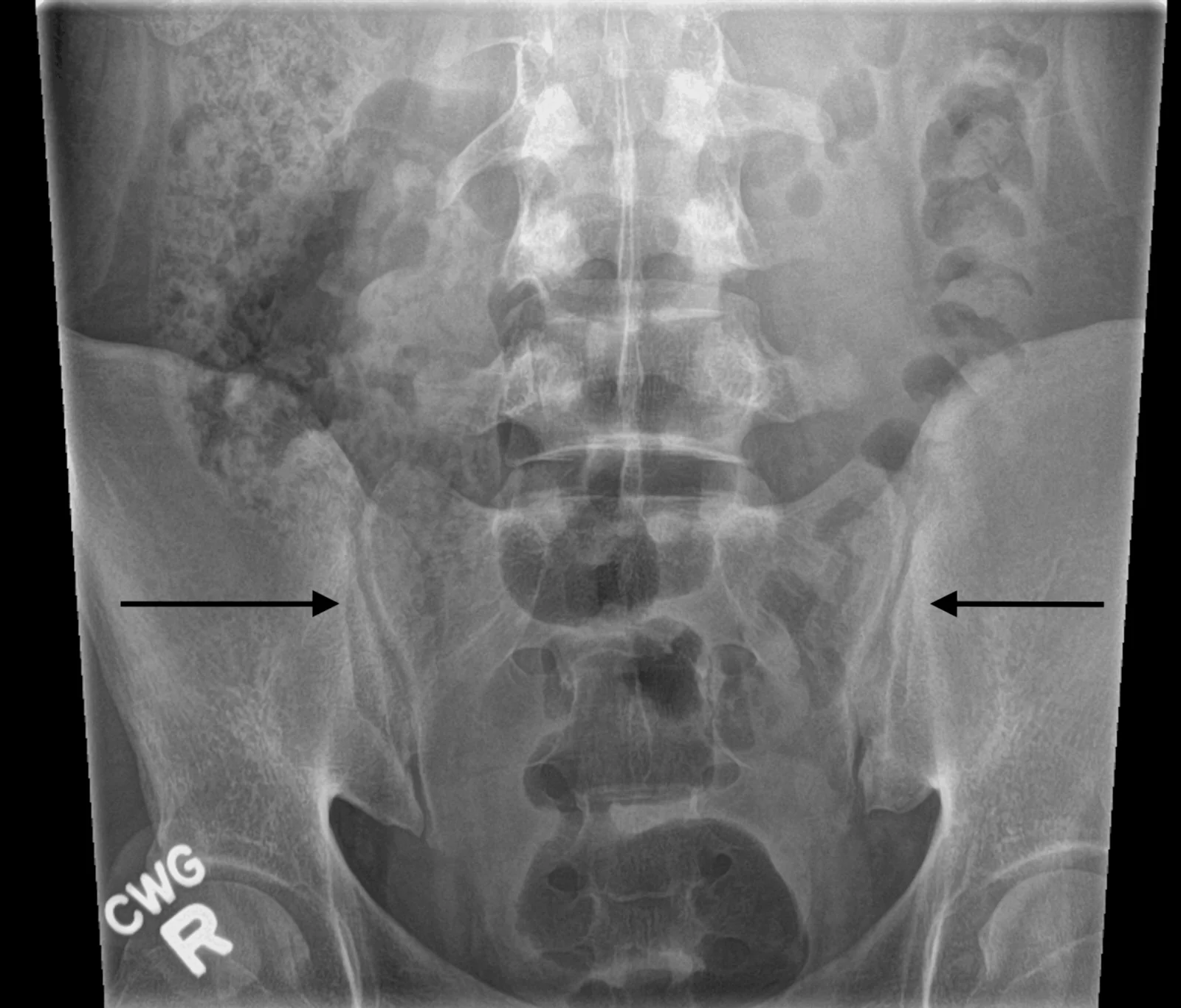
Anteroposterior dedicated sacroiliac joint radiograph. Bilateral sacroiliac joints (arrows) demonstrate normal joint space and cortical margins without radiographic evidence of sacroiliitis, erosion, or subchondral sclerosis.

Dermatologic findings

Clinical photographs of the bilateral lower extremities documented multiple ecchymotic and bruising lesions of varying sizes distributed across the thighs and lower legs bilaterally, without associated warmth, induration, or palpable purpura on physical examination. The lesions occurred without preceding trauma and were mildly tender. Per the available records, the patient did not establish care with dermatology, and dermatology consultation, dermoscopy, or skin biopsy were not performed or documented. As such, these lesions have not been formally evaluated to distinguish simple ecchymosis from an inflammatory or vasculitic process, and this represents a limitation in the current diagnostic evaluation; the photographs presented (Figures 2-3) should be interpreted as clinical documentation of gross appearance only, not as a substitute for dermatologic or histopathologic characterization.

**Figure 2:**
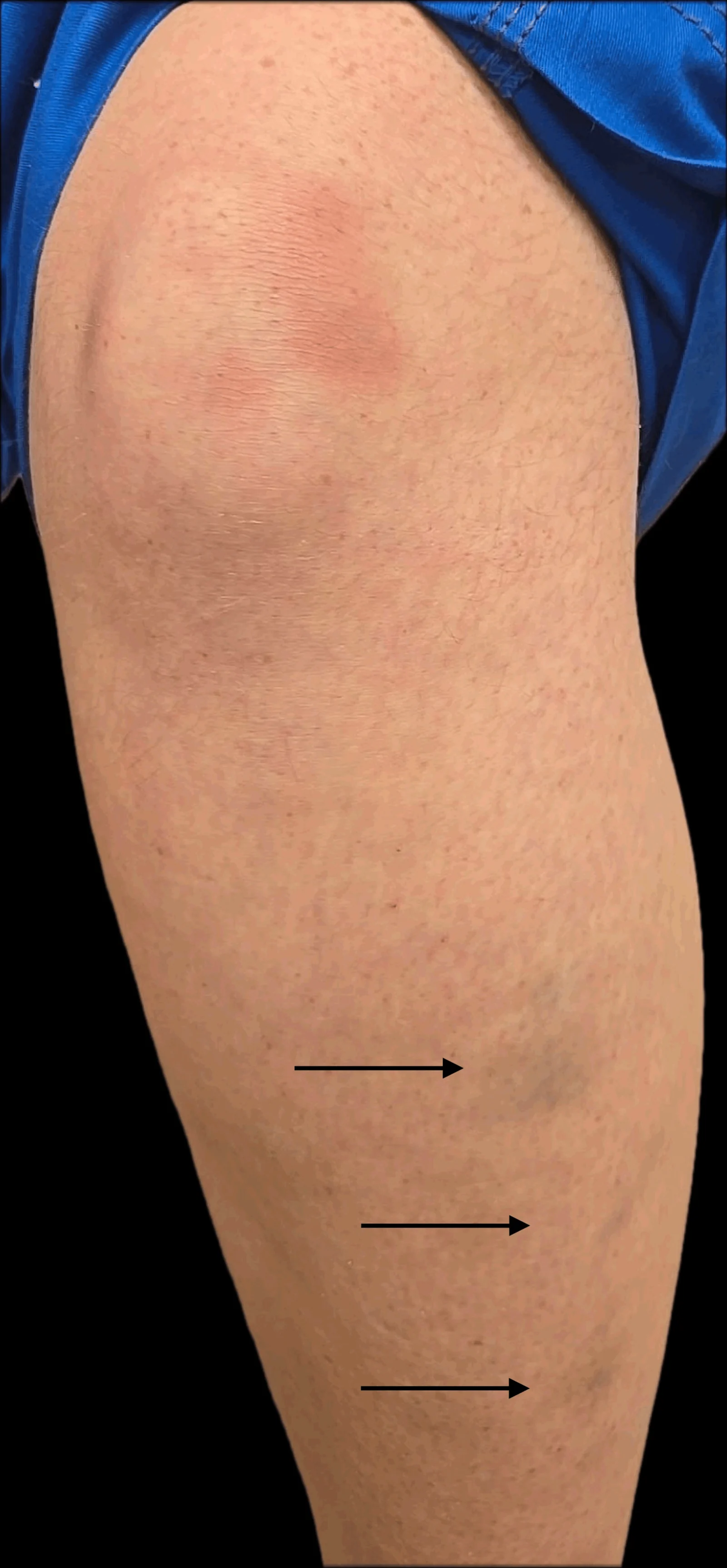
Left lower extremity demonstrating ecchymotic lesions (arrows) along the anterior shin and distal thigh, occurring in the absence of trauma.

**Figure 3:**
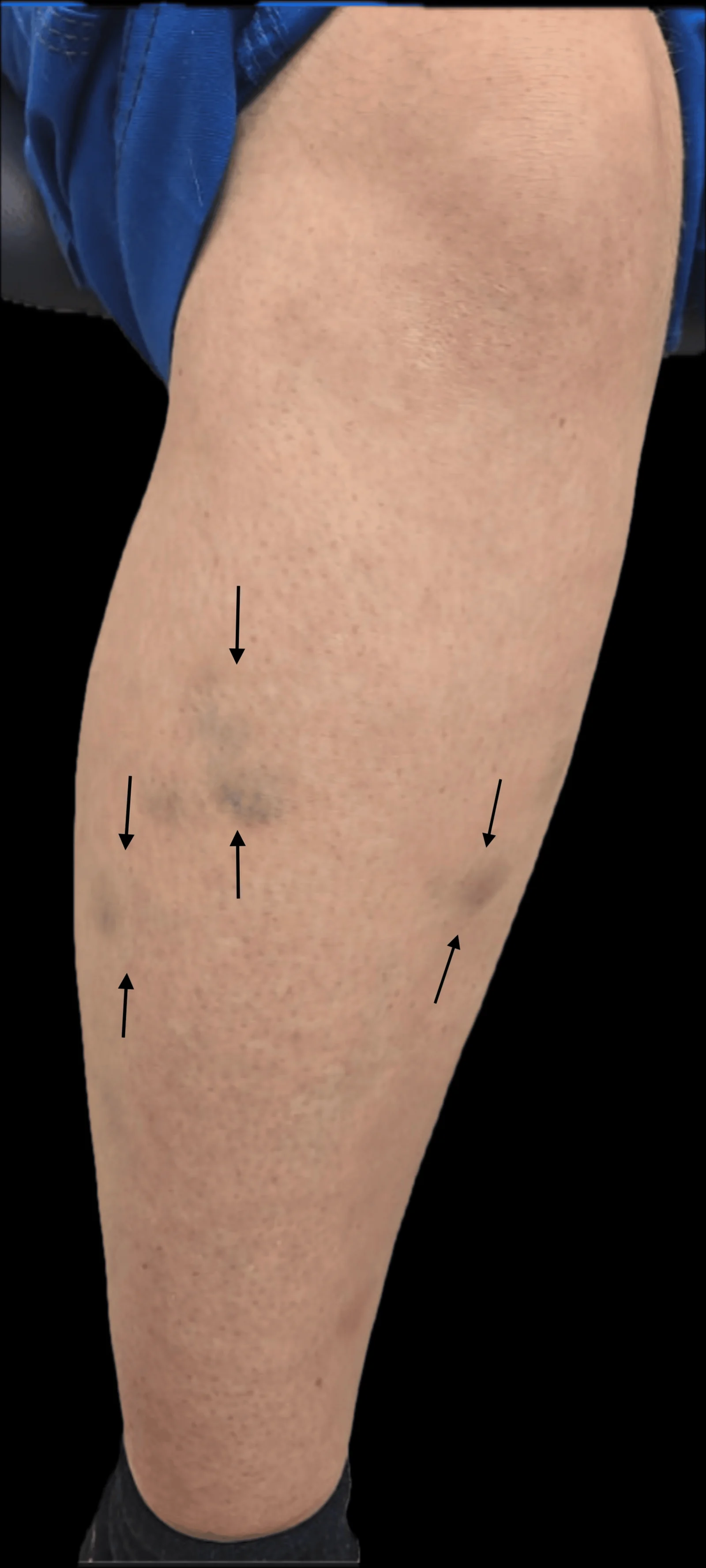
Right lower extremity demonstrating multiple ecchymotic lesions of varying size (arrows) distributed across the calf and mid-leg, with no associated erythema or induration.

Follow-up: internal medicine continuity visit (month 5 post-presentation)

At a follow-up continuity clinic visit (May 2026), the patient continued to report back pain as well as multiple ecchymoses and bruises without apparent cause. The clinical team engaged in a prolonged discussion regarding her previous findings and plan moving forward, including the possibility of psoriasis and psoriatic arthritis; further imaging was planned to assess for this. She also reported persistent migraine headache despite taking sumatriptan and amitriptyline. The team discussed Sjögren's syndrome-related hydration measures, as well as combining ondansetron (Zofran) with a possible NSAID prior to symptom onset and adjusting the timing of her medications, with plans to follow up with her neurologist for further escalation depending on her response. She understood the plan and all questions were answered.

Active problems at this visit included skin rash, arthralgia of both knees, leukopenia, menorrhagia with regular cycle, bilateral hip pain, bruising, and iron deficiency without anemia, given her normal hemoglobin on prior testing.

Follow-up: rheumatology consultation (month 6 post-presentation)

The patient subsequently completed formal rheumatology evaluation. Vital signs were as follows: blood pressure 111/68 mmHg, heart rate 66/min, temperature 98.1°F, oxygen saturation 98%, weight 152.8 lb, BMI 25.42, and pain score 2/10. General examination showed a well-appearing patient in no acute distress, with a normocephalic, atraumatic head; skin without visible rash, lesions, or nodules; no cyanosis or peripheral edema; non-labored breathing without wheezing or distress; and a normal neurologic exam (alert, oriented ×3, no focal deficits) with normal mood and affect. A formal 70-joint homunculus examination was performed and demonstrated mild tenderness at the left shoulder and bilateral knees and ankles, with all remaining joints (including wrists, hands, hips, and toes) recorded as normal without swelling, erythema, or deformity; the cervical spine and sacroiliac joints were also documented as normal on this exam.

She reported that mesalamine had been recently restarted under gastroenterology care for UC.

The rheumatology assessment identified inflammatory polyarthropathy as the primary diagnosis, with likely enthesitis at the bilateral ankles noted on clinical examination. The clinical picture-HLA-B27 positivity, history of UC, bilateral ankle enthesitis, and axial symptoms-was considered most consistent with IBD-associated spondyloarthropathy. Psoriatic arthritis remained on the differential pending further dermatologic evaluation. The treatment plan included escalation of therapy toward biologic agents, specifically tumor necrosis factor (TNF) inhibitors, with consideration of infliximab versus adalimumab given the patient's age, disease burden, and underlying IBD; specific dosing regimens had not yet been determined for these agents. Pre-biologic workup was initiated, including QuantiFERON-TB Gold, RheumAssure serologic panel, hepatic function testing, and renal function panel. Bilateral ankle radiographs were additionally ordered. Leukopenia was noted to have reportedly improved and was to be monitored. Follow-up was planned at four to six weeks.

Laboratory findings

Laboratory evaluation performed in the internal medicine clinic revealed persistent mild leukopenia, with WBC counts of 3.5-4.5 ×10³/µL across visits. On the rheumatology panel (June 2026), the absolute neutrophil count (2.5 ×10³/µL, reference 1.4-7.0) and absolute lymphocyte count (1.4 ×10³/µL, reference 0.7-3.1) were both within normal limits despite the mildly low total WBC. A peripheral blood smear was not documented as having been performed in the available records. Hemoglobin was stable and normal (13.0-13.6 g/dL), and platelet counts were repeatedly normal across all three available measurements (289, 293, and 308 ×10³/µL), which, per current documentation, is more consistent with an absence of immune thrombocytopenia than with a separate targeted immune thrombocytopenia (ITP) workup ruling it out. Medication review at the time of leukopenia workup included mesalamine, amitriptyline, meclizine, and sumatriptan; no thiopurine, methotrexate, or other agent classically associated with drug-induced leukopenia was documented in her medication history. A dedicated nutritional assessment specific to the leukopenia was not documented. The leukopenia was noted by the treating clinicians as "reportedly improved" on serial follow-up, with a plan to continue monitoring; no formal hematology consultation was documented in the available records.

Iron studies obtained in January 2026 showed ferritin 15.5 ng/mL (reference 11-306.8 ng/mL), serum iron 67 µg/dL, iron saturation 18% (reference 15-50%), and unsaturated iron-binding capacity 303 µg/dL (reference 155-355 µg/dL); calculated total iron-binding capacity was approximately 370 µg/dL. With hemoglobin and all iron indices within normal reference limits, these results do not support a diagnosis of either iron deficiency anemia or isolated iron deficiency at the time of testing. As an interpretive caveat, ferritin is an acute-phase reactant and can be falsely normal or elevated in the setting of chronic inflammation, which could theoretically mask coexisting iron deficiency; however, based on the concordant normal transferrin saturation and normal hemoglobin in this case, there is no laboratory evidence in the available records to support underlying iron deficiency.

Inflammatory markers were mildly elevated on CRP (4-11 mg/L across visits) with normal ESR (3-18 mm/hour across visits). Coagulation studies (PT 10.9 seconds, INR 1.0) were normal. However, as normal PT/INR do not exclude VWD or platelet-function disorders, we clarify that the available documentation states only that a panel "to rule out VWD and ITP," consisting of PT/INR, aPTT, CBC, ANCA panel, and iron panel, was sent and reported as unremarkable; individual von Willebrand factor antigen, von Willebrand factor activity, factor VIII activity, and platelet-function assay results are not itemized in the records available to us, and their specific values cannot be reported here. This represents a limitation of the current workup rather than a fully excluded diagnosis.

HLA-B27 was positive. Rheumatoid factor (RF) was negative (<14.0 units/mL), as was cyclic citrullinated peptide (CCP) antibody IgG/IgA (<20 units) and 14.3.3 eta protein (<0.20 ng/mL) on the RheumAssure panel. ANCA panel and Sjögren's antibodies (SS-A, SS-B) were negative. An extended autoimmune panel obtained by the primary care provider (December 2025), including chromatin, ribosomal P, centromere, Smith, RNP, anti-Scl-70, Jo-1, and dsDNA antibodies, was also negative, with negative hepatitis B/C serologies. QuantiFERON-TB Gold Plus was negative, which is relevant given that biologic therapy is under consideration. Laboratory findings are listed in Table 1.

**Table 1:** Laboratory findings at initial presentation and follow-up. ANCA, anti-neutrophil cytoplasmic antibody; ANC, absolute neutrophil count; ALC, absolute lymphocyte count; CCP, cyclic citrullinated peptide; CRP, C-reactive protein; ESR, erythrocyte sedimentation rate; HLA-B27, human leukocyte antigen B27; INR, international normalized ratio; MCV, mean corpuscular volume; PT, prothrombin time; RF, rheumatoid factor; UIBC, unsaturated iron-binding capacity; WBC, white blood cell count

Test	Result	Reference Range	Interpretation
WBC	3.5-4.5 ×10³/µL	4.0-11.0 ×10³/µL	Low (mild leukopenia)
ANC	2.5 ×10³/µL	1.4-7.0 ×10³/µL	Normal
ALC	1.4 ×10³/µL	0.7-3.1 ×10³/µL	Normal
Hemoglobin	13.0-13.6 g/dL	12.0-15.5 g/dL	Normal
Platelets	289-308 ×10³/µL	150-400 ×10³/µL	Normal (repeatedly)
MCV	95-96 fL	80-100 fL	Normal
Ferritin	15.5 ng/mL	11-306.8 ng/mL	Normal
Serum iron	67 µg/dL	50 - 212 ug/dL	Normal
Iron saturation	18%	15-50%	Normal
UIBC	303 µg/dL	155-355 µg/dL	Normal
ESR	3-18 mm/hour	0-20/0-32 mm/ hour	Normal
CRP	4-11 mg/L	<5-10 mg/L	Mildly elevated (visit-dependent)
PT	10.9 seconds	9.5-13.5 seconds	Normal
INR	1	0.8-1.1	Normal
vWF antigen/activity, factor VIII, platelet function	Not itemized in available records	-	Not reported
HLA-B27	Positive	Negative	Positive
RF	<14.0 units/mL	-	Negative
CCP Ab (IgG/IgA)	<20 units	-	Negative
14.3.3 eta	<0.20 ng/mL	-	Negative
ANCA panel	Unremarkable	Negative	Negative
Sjögren's Ab (SS-A/SS-B)	Negative	Negative	Normal
Extended ANA-related panel (chromatin, ribosomal P, centromere, Smith, RNP, Scl-70, Jo-1, dsDNA)	Negative	Negative	Negative
QuantiFERON-TB Gold Plus	Negative	Negative	Negative (pre-biologic screen)

## Discussion

Musculoskeletal manifestations are among the most common EIMs of IBD, occurring in both Crohn's disease and UC, and may occur independently of intestinal disease activity [1-3]. This patient's HLA-B27 positivity is consistent with the reported range of 25-78% among IBD patients with axial involvement, though this association is notably weaker than the >90% seen in idiopathic ankylosing spondylitis [6]. Her positive result functions as one supportive finding rather than a diagnostic one, in line with the broader literature. Similarly, her mildly elevated CRP with normal ESR mirrors previously reported observations that inflammatory markers correlate imperfectly with axial and entheseal disease activity in IBD-associated spondyloarthropathy [4], in contrast to peripheral arthritis patterns, which more frequently parallel bowel disease activity [1,2]. Her joint examination is more consistent with an axial/entheseal-predominant phenotype than the classic oligoarticular type 1 peripheral arthritis pattern described in the IBD literature, which typically involves acute, self-limited, asymmetric large-joint involvement paralleling flares [1-3]. This distinction is relevant because it shaped the working diagnosis toward axial spondyloarthropathy rather than peripheral enteropathic arthritis.

Her unremarkable sacroiliac radiographs similarly parallel a recognized limitation described in the literature: plain radiography has limited sensitivity for detecting early axial inflammatory changes, and normal radiographs do not exclude non-radiographic axial disease, which typically requires MRI and correlation with SpA classification criteria for further characterization [4,9]. Unlike cases in which MRI confirms active sacroiliitis, this patient's diagnosis remains a working clinical impression rather than a criteria-confirmed diagnosis, a distinction this report makes explicitly rather than implying imaging-based confirmation.

The co-occurrence of unexplained bruising, menorrhagia, and mild leukopenia in this patient is not a classically described EIM pattern and required a distinct, parallel hematologic evaluation. In contrast to isolated inflammatory arthropathy cases, her repeatedly normal platelet counts and coagulation studies argue against thrombocytopenia or a coagulation-factor deficiency as an explanation for her bleeding symptoms, though - unlike a case with complete von Willebrand factor and platelet-function testing - this could not be fully confirmed given the incomplete panel available in her records. Her leukopenia remained mild and stable without an identifiable medication or nutritional cause, distinguishing it from thiopurine- or methotrexate-associated cytopenias more commonly reported in IBD populations, as she had no exposure to either agent.

The differential diagnosis of psoriatic arthritis, raised given her recurrent rash, reflects the recognized overlap between enteropathic arthritis, axial SpA, and psoriatic arthritis within the broader seronegative spondyloarthropathy family [2,3]; unlike cases with active psoriatic skin or nail findings on examination, this patient had none documented, though the absence of formal dermatologic evaluation limits how confidently this diagnosis can be excluded compared to cases with dermatology-confirmed absence of disease.

Management of IBD-associated spondyloarthropathy is guided by both articular and intestinal disease burden. NSAIDs are first-line for peripheral and axial articular symptoms but warrant caution in active IBD given the risk of exacerbating intestinal inflammation [6,10]; here, no scheduled NSAID was added given her existing as-needed use and gastritis history, and mesalamine 1.2 g twice daily was restarted with gastroenterology. TNF inhibitors, including infliximab and adalimumab, are established for both UC and axial spondyloarthropathy and offer the advantage of treating both processes concurrently [7,11]; agent selection was under consideration with gastroenterology, though a specific joint decision-making process was not detailed in her records, and we emphasize that this escalation rests on a working rather than confirmed diagnosis. Pre-biologic screening (negative QuantiFERON-TB Gold Plus, RheumAssure panel, and hepatitis B/C serologies, with hepatic/renal function testing) was consistent with standard safety evaluation prior to TNF inhibitor therapy [11], though vaccination assessment was not documented. Gynecologic referral and ferrous sulfate 325 mg every other day were recommended for her menorrhagia and iron deficiency, though tolerance and follow-up response were not documented.

Taken together, this patient's presentation aligns with several well-described features of IBD-associated axial spondyloarthropathy (HLA-B27 positivity, disproportionately mild inflammatory markers, radiographically silent axial disease) while diverging from the classic peripheral arthritis pattern more typically described as paralleling bowel disease activity. The coexistence of unexplained hematologic findings further distinguishes this case from more straightforward EIM presentations and underscores the value of parallel, multidisciplinary evaluation, along with coordinated treatment planning across specialties, rather than diagnostic closure based on musculoskeletal findings alone.

## Conclusions

In a young woman with UC, inflammatory-pattern polyarthralgia with axial symptoms and HLA-B27 positivity supported a working diagnosis of IBD-associated seronegative spondyloarthropathy, despite only mildly elevated inflammatory markers and unremarkable sacroiliac radiographs. Concurrent unexplained bruising, menorrhagia, and leukopenia were not fully explained by targeted workup and should not be assumed secondary to her rheumatologic diagnosis. This case underscores two case-specific lessons: normal radiographs and mild inflammatory markers should not lower suspicion for axial spondyloarthropathy in HLA-B27-positive IBD patients, and unexplained hematologic findings in this setting warrant complete, dedicated evaluation rather than attribution to the primary rheumatologic or gastrointestinal diagnosis.
